# *Lamellodiscus louiseuzeti* n. sp. (Monopisthocotyla: Diplectanidae: Lamellodiscinae): a parasite of *Chaetodon hoefleri* (Teleostei, Chaetodontidae) with distinct attachment organ morphology

**DOI:** 10.1051/parasite/2026053

**Published:** 2026-10-02

**Authors:** Arfang Diamanka, Armando J. Cruz-Laufer, Sikhou Drame, Abdou Toure, Maarten P. M. Vanhove, Antoine Pariselle, Ngor Faye

**Affiliations:** 1 Laboratory of General Parasitology, Department of Animal Biology, Faculty of Sciences and Technologies, University Cheikh Anta Diop of Dakar BP: 5005 Dakar Senegal; 2 Research Group Zoology: Biodiversity & Toxicology, Centre for Environmental Sciences (CMK), Hasselt University 3590 Diepenbeek Belgium; 3 Systems Ecology and Resource Management Research Unit (SERM), Université libre de Bruxelles-ULB Brussels Belgium; 4 Freshwater Biology, Operational Directorate Natural Environment, Royal Belgian Institute of Natural Sciences 1000 Brussels Belgium; 5 Institute of Evolutionary Science of Montpellier (ISEM), Centre National de la Recherche Scientifique (CNRS), Université de Montpellier, Institut de Recherche pour le Développement (IRD) 34095 Montpellier France; 6 Biodiversity, Ecology and Genome Laboratory, Faculty of Sciences, Mohammed V University in Rabat 10000 Rabat Morocco

**Keywords:** Monogenea, Taxonomy, Phylogenetic analysis, *Lamellodiscus*, Senegal, West Africa

## Abstract

During a study on representatives of Diplectanidae (Monopisthocotyla) infecting *Chaetodon hoefleri*, a marine teleost from Senegal, we collected individuals belonging to a species of Lamellodiscinae, which showed a unique arrangement of the haptoral lamellodiscs, so far unreported in the literature, with the first lamella of the ventral lamellodisc open anteriorly, unlike the dorsal lamellodisc, which is closed. Due to this arrangement, we describe the species *Lamellodiscus louiseuzeti* n. sp.. Apart from the morphology of the lamellodiscs, the new species differs from its congeners by having a two-part male copulatory organ; one part featuring a well-developed distal hook and one, morphologically very variable, exhibiting a distal tongue-shaped part. Phylogenetic analyses of a concatenated alignment of partial *18S* rDNA, ITS1, and *COI* sequences (1,315 bp), and a separate *28S* rDNA alignment (882 bp) of this new species and other species of Lamellodiscinae with available DNA sequence data resulted in phylogenetic trees with congruent topologies that indicated three main lineages. These clades correspond to the morphological groups of *Lamellodiscus* suggested in previous studies. Based on phylogenetic analyses, *L. louiseuzeti* n. sp. forms a well-supported group with other species of *Lamellodiscus.* However, its internal phylogenetic position within the genus remains unresolved. We opted to consider *L. louiseuzeti* n. sp. a member of *Lamellodiscus*. We also emended the generic diagnosis to incorporate the new species and propose a new morphological group: Heterolamellae.

## Abstract (fr)

Lors d’une étude sur des représentants de la famille des Diplectanidae (Monopisthocotyla) parasitant *Chaetodon hoefleri*, un téléostéen marin du Sénégal, nous avons récolté des individus appartenant à une espèce de Lamellodiscinae. Ces spécimens présentaient une disposition unique des lamellodisques du hapteur, jamais décrite auparavant dans la littérature, caractérisée par une première lamelle du lamellodisque ventral ouverte antérieurement, contrairement à celle du lamellodisque dorsal qui est fermée. En raison de cette disposition, nous décrivons l’espèce *Lamellodiscus louiseuzeti* n. sp.. Outre la morphologie des lamellodisques, cette nouvelle espèce se distingue de ses congénères par un organe copulateur mâle composé de deux parties, l’une dotée d’un crochet distal bien développé et l’autre, morphologiquement très variable, présentant une partie distale en forme de languette. Les analyses phylogénétiques, fondées sur un alignement concaténé de séquences partielles d’ADNr *18S*, d’ITS1 et de *COI* (1315 pb) ainsi que sur un alignement distinct d’ADNr *28S* (882 pb) incluant cette nouvelle espèce et d’autres Lamellodiscinae dont les séquences d’ADN sont disponibles, ont produit des arbres phylogénétiques aux topologies congruentes, révélant trois lignées principales. Ces clades correspondent aux groupes morphologiques de *Lamellodiscus* suggérés dans des études antérieures. D’après ces analyses, *L. louiseuzeti* n. sp. forme un groupe bien soutenu avec d’autres espèces de *Lamellodiscus*, bien que sa position phylogénétique interne au sein du genre reste indéterminée. Nous avons choisi de classer *L. louiseuzeti* n. sp. dans le genre *Lamellodiscus*. Nous avons également amendé la diagnose générique pour y intégrer cette nouvelle espèce et proposons un nouveau groupe morphologique : Heterolamellae.

## Introduction

Lamellodiscinae Oliver, 1969 (Monopisthocotyla, Diplectanidae) is a subfamily of parasitic flatworms living on the gills of teleost fishes. Lamellodiscines are monopisthocotylans, which represent one of the most species-rich [12] groups of fish ectoparasites. These parasites are often host-specific, which can provide detailed insight into co-evolutionary processes and host switching [18, 49]. Therefore, studying the evolutionary history and ecology of members of Lamellodiscinae may lead to a better understanding of the evolutionary adaptations and diversification of parasites in marine ecosystems [18].

Originally erected by Oliver [54], the subfamily currently encompasses four valid genera: *Lamellodiscus* Johnston & Tiegs, 1922, *Protolamellodiscus* Oliver, 1969, *Calydiscoides* Young, 1969, and *Telegamatrix* Ramalingam, 1955 [55]. Species of Diplectanidae Monticelli, 1903 possess an opisthohaptor with one or two lamellodiscs formed of concentric rows of paired or unpaired lamellae and two pairs of anchors joined by three transverse bars (two dorsolateral bars and one ventral median bar) [54]. The subfamily originally encompassed *Lamellodiscus* Johnston & Tiegs, 1922, *Protolamellodiscus* Oliver, 1969, and *Furnestinia* Euzet & Audouin, 1959 (now a synonym of *Lamellodiscus* [15]). Oliver [55] later added *Calydiscoides* Young, 1969 and *Telegamatrix* Ramalingam, 1955, after which the list of genera included in Lamellodiscinae has remained unchanged. While these genera are primarily differentiated by the specific arrangement and morphology of the concentric lamellae on their attachment organs (lamellodiscs) [54, 61], most other diagnostic traits overlap across the subfamily [55]. This morphological homogeneity has led authors to question the monophyly of *Lamellodiscus*, the most species-rich genus with 61 valid species [76], suggesting that it may serve as a “catch-all” group for species lacking conspicuous derived characters [15, 40].

This taxonomic uncertainty is further compounded by the available genetic data. To date, 32 species of *Lamellodiscus* have been sequenced: fragments of *18S* ribosomal DNA (rDNA) have been sequenced for 26 species, *28S* rDNA for 11 species, cytochrome *c* oxidase subunit I mitochondrial DNA (*COI* mtDNA) for seven species (Table 2), and internal transcribed spacer 1 (ITS1) for 13 species (see Table 2 and [33]). Some authors have also questioned the validity of certain species of *Lamellodiscus* based on these molecular data. For instance, *L. coronatus* Euzet & Oliver, 1966 was suggested to be a potential synonym of *L. furcosus* Euzet & Oliver, 1966 based on phylogenetic analyses [59]. Similar concerns were raised for *L. virgula* Euzet & Oliver, 1967 and *L. obeliae* Oliver, 1973 as partial nuclear DNA sequences of ITS1 differed by only 0.27% [59]. These discordances between the morphological characters used to delimit species and species groups and some molecular data [47, 58, 74] highlight the need to reassess the congruence between morphological similarity of species and their evolutionary relatedness.

In this study, we describe a new species of *Lamellodiscus* from *Chaetodon hoefleri* Steindachner, 1881 using morphological and molecular data. The discovery of a species of *Lamellodiscus* from this host is remarkable as previous studies found that species of *Lamellodiscus* parasitise mainly sparid and lethrinid fishes, with few cases reported from centracanthids, pomacanthids, and pomacentrids [4, 39, 43, 53]. No study to date has reported species of *Lamellodiscus* from a chaetodontid host, making this record a new host association that raises immediate questions regarding the host specificity and intergeneric relationships of species of *Lamellodiscus*. The new species also presents a hitherto unknown type of attachment organ configuration for lamellodiscines.

## Materials and methods

### Ethical standards

The authors confirm that all applicable institutional, national, and international guidelines for the care and use of animals were followed.

### Sample collection and morphological analysis

Fifty-three specimens of *Chaetodon hoefleri* were collected from the Soumbédioune fish market, near Dakar (14°40′41″N, 17°27′34″W), Senegal, in December 2009 and, more recently, supplementary samples in March 2025. The fish were identified using keys provided by Blache [6] and dissected immediately or frozen until examination. Gill arches were removed, placed in separate Petri dishes containing seawater, and examined for parasites under a stereomicroscope. Monopisthocotylan flatworms were detached from the gills with a strong water current and transferred to a dish containing filtered seawater. Some parasites were slightly compressed beneath slides and coverslips and examined alive using an optical microscope. Others were fixed in hot 70% ethanol and stained with acetocarmine before mounting in Canada balsam. Other specimens were mounted in Malmberg’s medium (ammonium picrate-glycerol fluid) [44], then covered with a round coverslip and sealed with Glyceel [5].

Drawings were made using an Orthoplan microscope (Leica Microsystems, Wetzlar, Germany) equipped with a drawing tube, then scanned and redrawn with CorelDRAW Graphics Suite X4 (Corel Corporation, Ottawa, ON, Canada). Measurements of specimens mounted in Malmberg’s medium were obtained using a DM2500 Leica microscope, a DFC425 Leica digital camera, and Leica Application Software v 3.6. Measurements are provided in μm as the mean ± standard deviation followed in parentheses by the range and the number (*n*) of observations. The terminology and measurements of the sclerotised structures in the main attachment organ were applied according to Neifar *et al.* [52]. The rows of lamellae were numbered sequentially from the innermost to the outermost. Type material was deposited in the collections of the Muséum national d’Histoire naturelle (MNHN), Paris, France and of the Invertebrate Collection of Hasselt University (HU), Hasselt, Belgium.

### DNA extraction and amplification

The DNA extraction of a single complete specimen was performed in a UV cabinet. All utensils were sterilised and UV-irradiated before usage. The extraction was completed according to a previously published protocol [37], using enzymatic digestion with proteinase K (55 °C, 400 rpm, 1 h) and DNA precipitation through ethanol. We amplified and sequenced three partial nuclear ribosomal markers, including a fragment of the large subunit ribosomal DNA (*28S*), a fragment of the small subunit (*18S*) and the internal transcribed spacer 1 (ITS1), and a fragment of the mitochondrial gene coding for the cytochrome *c* oxidase subunit 1 protein (*COI*). The exact composition of the reaction mixes and reaction protocols can be seen in Table 1. The PCR products were purified using a GeneJET PCR Purification Kit (Thermo Fisher Scientific, Waltham, MA, USA). Sanger sequencing was performed using the same primers as in the initial amplification and was outsourced to Macrogen Europe (Amsterdam, Netherlands). Sequence quality was checked visually through the sequencing chromatograms in Geneious Prime v2025.0.2 (Biomatters, Auckland, New Zealand). The sequences were uploaded to GenBank under accession numbers: PZ390892 (*28S*), PZ390890 (*18S-*ITS1), and PZ387863 (*COI*).

**Table 1:** Overview of primers and PCR settings. The total volume of each reaction was 25 μL.

Region	Forward primer	Reverse primer	Reference	Reaction mix	PCR conditions
*28S*	C1: 5′–ACCCGCTGAATTTAAGCAT–3′	D2: 5′–TGGTCCGTGTTTCAAGAC–3′	[28]	12.5 μL 2× Mango Mix (Meridian Bioscience, Cincinnati, OH, USA) 0.5 μL MgCl_2_ 1.25 μL of each primer 7.5 μL ddH_2_0 2.0 μL DNA sample	1) 94 °C, 2 min 2) 94 °C, 20 s 3) 56 °C, 30 s 4) 72 °C, 1:30 min 39× repeat: 2–4 5) 72 °C, 10 min
*18S* + ITS1	S1: 5′–ATTCCGATAACGAACGAGACT–3′	IR8: 5′–GCAGCTGCGTTCTTCATCGA–3′	[46] [69]	12.5 μL 2× Mango Mix 0.5 μL MgCl_2_ 1.25 μL of each primer 4.5 μL ddH_2_0 5.0 μL DNA sample	1) 94 °C, 2 min 2) 94 °C, 1 min 3) 53 °C, 1 min 4) 72 °C, 1:30 min 39× repeat: 2–4 5) 72 °C, 10 min
*COI*	ASmit1: 5′–TTTTTTGGGCATCCTGAGGTTTAT–3′	Cox1_Schisto_3: 5′–TCTTTRGATCATAAGCG–3′ ASmit2: 5′–TAAAGAAAGAACATA ATGAAAATG–3′	[41] [42]	12.5 μL 2× Mango Mix 0.5 μL MgCl_2_ 1.0 μL of each primer 0.25 μL bovine serum albumin (BSA) 6.75 μL ddH_2_0 2.0 μL DNA sample	1) 94 °C, 5 min 2) 94 °C, 1 min First PCR: 3) 45 °C (first PCR) or 50 °C (second PCR), 1 min 4) 72 °C, 1 min 39× repeat: 2–4 5) 72 °C, 7 min

### Phylogenetic analyses

To infer the evolutionary history of the new species, we assembled multiple sequence alignments of *28S*, *18S*, ITS1 and *COI*, combining the sequences generated during this study with sequences accessed on NCBI GenBank for species of Lamellodiscinae, which at the time of writing included species of *Lamellodiscus* and a single species of *Protolamellodiscus* (Table 2). As an outgroup (for rooting), we also included *Dolicirroplectanum lacustre* (Thurston & Paperna, 1969), *Paradiplectanum sillagonum* (Tripathi, 1959), and *Pseudorhabdosynochus grouperi* (Bu, Leong, Wong, Woo & Foo, 1999), which are species belonging to Diplectaninae Monticelli, 1903, another subfamily of Diplectanidae.

**Table 2:** Specimen data for DNA sequences used for phylogenetic reconstruction including species of Lamellodiscinae and three additional species (*Dolicirroplectanum lacustre, Paradiplectanum sillagonum, Pseudorhabdosynochus grouperi*) used as outgroup. The data include host species unique identifier (ID) of specimens, GenBank accession numbers, and references.

Species	ID	Voucher	Host	*28S*	*18S*	ITS1	*COI*	Reference
*L. acanthopagri* Roubal, 1981			unknown	DQ054822	–	–	–	[79]
*L. baeri* Oliver, 1974			*Pagrus pagrus* (L., 1758)	–	AY038187	–	–	[18]
*L. bidens* Euzet, 1984			*Diplodus puntazzo* (Walbaum, 1792)	–	AY038188	–	–	[18]
*L. chin* Nitta, 2021	1		*Acanthopagrus sivicolus* Akazaki, 1962	LC565448	–	–	–	[53]
*L. chin*	2		*A. sivicolus*	LC565449	–	–	–	[53]
*L. confusus* Amine, Euzet & Kechemir-Issad, 2007		Co1Sa2_272	*D. sargus* (L., 1758) or *D. vulgaris* (Geoffroy Saint-Hilaire, 1817)	–	JF427643	JF427657	JF427650	[59]
*L. coronatus* Euzet & Oliver, 1966			*D.* sp.	–	AY038189	–	–	[18]
*L. diplodicus* Bayoumy, 2003		Di1Dv9_291	*D. vulgaris*	–	–	–	JF427654	[59]
*L. drummondi* Euzet & Oliver, 1967			*Pagellus acarne* (Risso, 1827)	–	AJ276441	AJ276445	–	[17]
*L. echeneis* (Wagener, 1857)	1		*Sparus aurata* L., 1758	–	AF294953	–	–	[16]
*L. echeneis*	2		*S. aurata*	–	JX090091	JX090091	JX090031	[51]
*L. egusai* Nitta, 2021	1		*A. schlegelii* (Bleeker, 1854)	EF100561	–	–	–	[78]
*L. egusai*	2		*A. latus* (Houttuyn, 1782)	FJ767865	–	–	–	[71]
*L. egusai*	3		unknown	–	EU836236	–	–	[72]
*L. elegans* Bychowsky, 1957	1	L._eleDS	*D. sargus*	–	JF427626	–	–	[59]
*L. elegans*	2	L._eleDV	*D. vulgaris*	–	JF427627	–	–	[59]
*L. elegans*	3	L._eleDA	*D. sargus* or *D. vulgaris*	–	JF427628	–	–	[59]
*L. elegans*	4	ELEDs3_266	*D. sargus*	–	JF427636	–	–	[59]
*L. elegans*	5		*D. sargus*	–	AF294956	–	–	[15]
*L. ergensi* Euzet & Oliver, 1966	1	L._erg2	*D. sargus* or *D. vulgaris*	–	JF427629	–	–	[59]
*L. ergensi*	2	Er1Ds3_242	*D. sargus*	–		–	JF427653	[59]
*L. ergensi*	3		*D.* sp.	–	AY038190	–	–	[18]
*L. erythrini* Euzet & Oliver, 1966			*Pagellus erythrinus* (L., 1758)	–	AJ276440	AJ276444	–	[17]
*L. falcus* Amine, Euzet & Kechemir-Issad, 2006	1	F1	*D. puntazzo*	–	KC470292	KC470298	–	[67]
*L. falcus*	2	F2	*D. puntazzo* (Walbaum, 1792	–	KC470293	KC470298	–	[67]
*L. falcus*	3	F3	*D. puntazzo*	–	KC470294	KC470298	–	[67]
*L. falcus*	4	Fa1Ds7_223	*D. sargus*	–	JF427640	JF427656	–	[59]
*L. fraternus* Bychowsky, 1957			*D. annularis* (L., 1758) or *D. vulgaris*	–	AY038191	–	–	[18]
*L. furcosus* Euzet & Oliver, 1966	1	Fu3Ds5_238	*D. sargus*	–	JF427642	–	JF427647	[59]
*L. furcosus*	2	Fu2Ds5_239	*D. sargus*	–	JF427644	–	JF427646	[59]
*L. furcosus*	3	Fu1Ds5_261	*D. sargus*	–	–	–	JF427645	[59]
*L. furcosus*	4		*D. annularis* or *D. sargus*	–	AY038192	–	–	[18]
*L. gracilis* Euzet & Oliver, 1966			*D. annularis*, *D. sargus*, or *Oblada melanura* (L., 1758)	–	AY038193	–	–	[18]
*L. hilii* Euzet, 1984			*D. puntazzo*	–	AY038194	–	–	[18]
*L. ignoratus* Palombi, 1943	1	L._ignDV	*D. vulgaris*	–	JF427630	–	–	[59]
*L. ignoratus*	2	L._ignDS2	*D. sargus*	–	JF427631	–	–	[59]
*L. ignoratus*	3	L._ignDS	*D. sargus*	–	JF427632	–	–	[59]
*L. ignoratus*	4	L._ignDP	*D. sargus* or *D. vulgaris*	–	JF427633	–	–	[59]
*L. ignoratus*	5	L._ignLM	*D. sargus* or *D. vulgaris*	–	JF427634	–	–	[59]
*L. ignoratus*	6	L._ignSS	*D. sargus* or *D. vulgaris*	–	JF427635	–	–	[59]
*L. ignoratus*	7	Ig2Ds7_233	*D. sargus*	–	JF427641	JF427658	JF427651	[33, 59]
*L. ignoratus*	8	Ig2Ds1_209	*D. sargus*	–	–	–	JF427655	[33, 59]
*L. ignoratus*	9	L._ignDS4	*D. sargus*	–	–	EU259028	–	[59]
*L. ignoratus*	10	L._ignDS5	*D. sargus*	–	–	EU259029	–	[33]
*L. ignoratus*	11	L._ignDS6	*D. sargus*	–	–	EU259030	–	[33]
*L. ignoratus*	12	L._ignDV3	*D. vulgaris*	–	–	EU259031	–	[33]
*L. ignoratus*	13	L._ignDV4	*D. vulgaris*	–	–	EU259032	–	[33]
*L. ignoratus*	14		*Sarpa salpa* (L., 1758)	–	AF294957	–	–	[15]
*L. neifari*	2	Ne1Ds6_252	*D. sargus*	–	JF427638	JF427659	–	[59]
*L. neifari* Amine, Euzet & Kechemir-Issad, 2006	1	Ne1Ds6_240	*D. sargus*	–	JF427637	JF427660	JF427648	[59]
*L. neifari*	3	Ne1Ds6_249	*D. sargus*	–	JF427639	JF427661	JF427649	[59]
*L. pagrosomi* Murray, 1931	1		*Pagrus major* (Temminck & Schlegel, 1843)	EF100562	–	–	–	[78]
*L. pagrosomi*	2		*Parargyrops edita* Tanaka, 1916	KY640620	–	–	–	[11]
*L. pagrosomi*	3		unknown	–	EU836235	–	–	[72]
*L. parisi* Oliver, 1969			*Sarpa salpa*	–	AY038198	–	–	[18]
*L. spari* Zhukov, 1970	1		unknown	DQ054823		–	–	[70]
*L. spari*	2		*A. schlegelii*	LC565450	–	–	–	[53]
*L. takitai* Ogawa & Egusa, 1978	1		*A. schlegelii*	LC565451	–	–	–	[53]
*L. theroni* Amine, Euzet & Kechemir-Issad, 2007	1	T1	*D. puntazzo*	–	KC470295	KC470301	–	[67]
*L. theroni*	2	T2	*D. puntazzo*	–	KC470296	KC470302	–	[67]
*L. theroni*	3	T3	*D. puntazzo*	–	KC470297	KC470303	–	[67]
***L. louiseuzeti* n. sp.**			***Chaetodon hoefleri* Steindachner, 1881**	**PZ390892**	**PZ390890**		**PZ387863**	**This study**
*L. verberis* Euzet & Oliver, 1967			*L. mormyrus*	–	AF294955	–	–	[15]
*L. virgula*	2		*Pagellus bogaraveo* (Brünnich, 1768)	–	AJ276443	AJ276447	–	[17]
*L. virgula* Euzet & Oliver, 1967	1		*P. acarne*	–	AJ276442	AJ276446	–	[17]
*Lamellodiscus abbreviatus* Sanfilippo, 1978			*D. sargus* or *D. vulgaris*	–	JF427625	–	–	[59]
*Dolicirroplectanum lacustre* (Thurston & Paperna, 1969)			*Lates niloticus* (L., 1758)	MK937579	MK937576	MK937576	MK908186	[38]
*Paradiplectanum sillagonum* (Tripathi, 1959)			*Sillago sihama* (Forsskål, 1775)	AY553626	AY553617	–	–	[77]
*Pseudorhabdosynochus grouperi* (Bu, Leong, Wong, Woo & Foo, 1999)			*Epinephelus coioides* (Hamilton, 1822)	AY553628	AY553618	–	–	[77]

We aligned rDNA sequences using L-INS-i in MAFFT v.7.409 [35] and removed poorly aligned positions and divergent regions using the options for less stringent parameters (options: −b2 = <minimum> − b3 = 10 − b4 = 5 − b5 = h) in Gblocks v.0.91b [73]. For *COI*, sequences were aligned in MACSE v2.06 [63, 64] using the algorithms trimNonHomologousFragments, alignSequences, and TrimSequenceAlignment under default parameters and the Flatworm Mitochondrial Code.

We estimated tree topologies through maximum likelihood (ML) and Bayesian inference (BI) methods using IQ-Tree v2.2.26 [50] and MrBayes v3.2.7 [66], respectively. Because of the minimal overlap in terms of species and specimens between the nuclear and mitochondrial loci of species of *Lamellodiscus*, we opted to concatenate only the *18S*, ITS1, and *COI* alignments and analyse the *28S* sequences separately. For the former, we rendered ML trees for each region separately and checked for congruence of the tree topologies using a Congruence Among Distance Matrices (CADM) test [7] as implemented in the package ape v.5.3 [56] in R v.4.5.0 [60].

The alignments were partitioned by locus. For ML and BI, we selected the substitution models for each partition according to the Bayesian information criterion (BIC) as suggested by ModelFinder in IQ-Tree [34] using partition merging [10] (Table 2). For ML, *COI* was modelled using codon models in ModelFinder. We estimated branch support values using both ultrafast bootstrap approximation (UF-Boot) [29] and Shimodaira–Hasegawa-like approximate likelihood ratio tests (SH-aLRT) [26] with 10,000 replicates. To minimise false positives, UF-Boot was estimated by resampling partitions as well as sites within resampled partitions [25, 68]. For BI analyses, model selection in ModelFinder was restricted to models implemented in MrBayes (Table 2) and, for *COI*, sequences were additionally partitioned by codon position. We used two runs and four chains of Metropolis-coupled Markov chain Monte Carlo [(MC)^3^] iterations with calculation conducted in parallel to optimise runtime [3]. We ran 20 million generations with a burn-in fraction of 0.25 and sampled the trees every 1,000^th^ generation. We checked convergence criteria by assessing the average standard deviation of split frequencies (<0.01 in all datasets) and the effective sample size (>200) with Tracer v1.7 [62]. Phylogenetic trees were visualised with ggplot2 v4.0.0 [75] and ggtree v3.16.3 [80, 81] in R.

## Results

Class: Monopisthocotyla Brabec, Salomaki, Scholz & Kuchta, 2023

Order: Dactylogyridea Bychowsky, 1937

Family: Diplectanidae Monticelli, 1903

Subfamily: Lamellodiscinae Oliver, 1969

### *Lamellodiscus* Johnston & Tiegs, 1922

#### Emended diagnosis (based on Oliver [55])

Body flattened dorso-ventrally. Three pairs of cephalic glands on each side of pharynx, both with ducts leading to three lateral head organs. Ocular spots present (two pairs) or absent. Deferent canal may or may not surround left digestive caecum. Mouth sub-terminal and ventral in cephalic region. Pharynx spherical, located on midline, posterior to eyespots. Oesophagus present. Two intestinal caeca simple, terminate blindly before haptoral peduncle. Testis subspherical, intercaecal, in anterior region of posterior half of body. Vas deferens originating on left side of testis, not encircling sinistral caecum, and enlarging to form one seminal vesicle. Prostatic reservoir pyriform, sometimes with two distinct regions, anterior to male copulatory organ. Male copulatory organ generally consisting of a more or less complex structure with accessory part(s). Vagina present; vaginal opening sinistral, exceptionally dextral. Tetrahedral eggs with a long polar filament. Oncomiracidium with four pairs of protonephridia. Haptor well-differentiated, with three interconnected transverse bars (two dorsal lateral bars and one ventral median bar); two pairs of anchors (each consisting of a dorsal anchor and a ventral anchor) arranged at the outer end of dorsal lateral transverse bar on the same side. Two lamellodiscs consisting of 10 rows of lamellae, each row consisting of paired or unpaired elements; terminal rows usually unpaired; if unpaired, row 10 curved like an arc, but can also be paired; row 1, or sometimes rows 1–2 forming a closed ring, but can also be open; other lamellae either paired or, if unpaired, more or less incurved in centre. The arrangement of lamellae may be symmetrical or asymmetrical. Fourteen (14) marginal hooks. Parasites of marine or brackish-water teleost fishes, mostly sparids and lethrinids, occasionally of centracanthids, chaetodontids, pomacanthids, and pomacentrids.

#### Remarks

Johnston and Tiegs [31] created *Lamellodiscus* to encompass diplectanids with accessory discs consisting of rows of “single pairs of laterally-elongated lamellae”. The generic diagnosis was later emended by Oliver [54, 55] to discriminate species of *Lamellodiscus* from the other genera included in the newly created Lamellodiscinae. Specifically, the arrangement of the rows of lamellae of the lamellodiscs was specified as rows 1 (sometimes 1 and 2) and 10 consisting of unpaired elements and the remaining rows consisting of paired ones. At the same time, the second emendation [55] was followed by remarks concerning morphological groups of species of *Lamellodiscus*. Several members of one of these groups, the “*ignoratus*” group, present more unpaired rows of lamellae than specified in the diagnosis, although these single elements are still incurved at the centre, where the paired elements are separated in those species that strictly adhere to the generic diagnosis. Furthermore, the first (or first two) rows of lamellae were defined as heart-shaped (“cordiform”) in the second emended diagnosis, implicitly suggesting that these lamellae form closed rings. The new species described in the present study (see below) exhibits an “open” row 1, i.e., with a gap in the lamella, in one of the lamellodiscs. Similarly, row 10 was defined as arc-shaped. However, both *L. echeneis* (Wagener, 1857) (formerly *Furnestinia echeneis* Euzet & Audouin, 1959) and the new species described in the present study (see below) possess a row 10 consisting of paired elements. Therefore, to accommodate these differences, we broadened the diagnosis of *Lamellodiscus*.

### *Lamellodiscus louiseuzeti* n. sp. Diamanka, Drame & Pariselle

urn:lsid:zoobank.org:act:2E2E9A99-9296-4B9D-AC0C-33DFE823003E

**Type host**: *Chaetodon hoefleri* Steindachner, 1881, Teleostei, Chaetodontidae.

**Infection site**: between secondary gill lamellae.

**Type locality**: Soumbédioune fish market (14°40′41.73″N, 17°27′34.31″W), Dakar, Senegal.

**Prevalence**: 100%, 53 infected of 53 fish examined.

**Intensity**: 12 (954 parasite specimens)

**Holotype**: MNHN Paris, No. MNHN-HE-131YT.

**Paratypes**: 2 at MNHN Paris, No. MNHN-HE-131YT; 3 at Invertebrate Collection of Hasselt University, HU 1133–1135.

**Etymology**: The species epithet was given to honour the late Prof. Louis Euzet, who was the first to discover the species during a research stay in Dakar.

**Note**: The authors of the new taxa are different from the authors of this paper: Article 50.1 and recommendation 50A of the International Code of Zoological Nomenclature [30].

**Description** (based on 30 flattened specimens) (Fig. 1) (measurements in μm).

**Figure 1:**
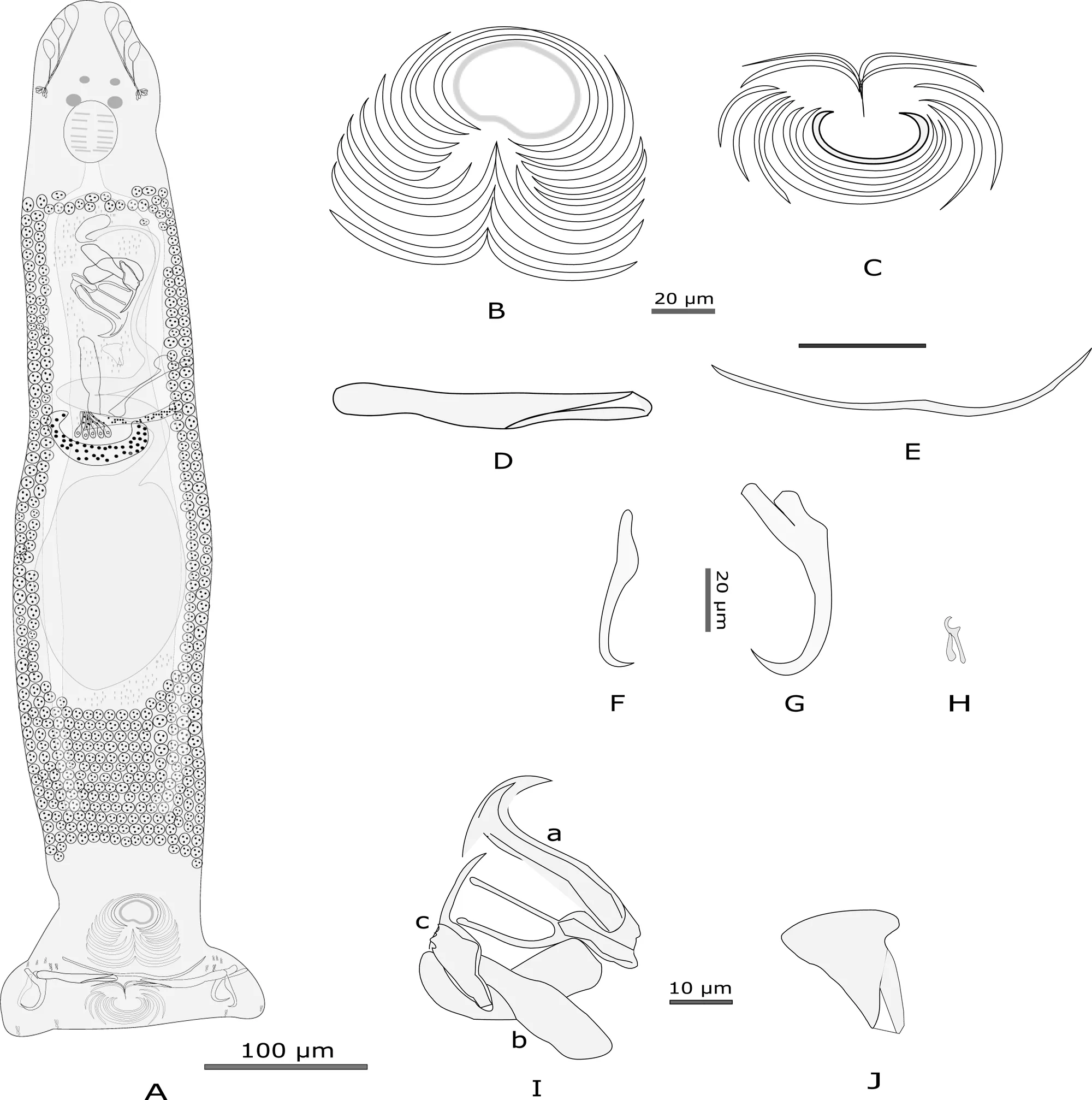
*Lamellodiscus louiseuzeti* n. sp. A Composite drawing of live specimen, ventral view. Haptoral sclerotised pieces: B dorsal lamellodisc, C ventral lamellodisc with 10 concentric lamellae, D dorsal bar, E ventral bar, F dorsal anchor, G ventral anchor, H marginal hook, I male copulatory organ, J oval sclerotised rough plate. Scale bars: A = 100 μm; B–G = 20 μm; H–J = 10 μm.

Adults 774 ± 65 (688–962, *n* = 30) long including haptor; maximum width 184 ± 25 (134–259, *n* = 30) at level of ovary (Fig. 1A). Haptor, well-differentiated and broader than body, 197 ± 21 (162–237, *n* = 30) wide. Two morphologically different lamellodiscs with 10 concentric lamellae (Figs. 1A–1C). Dorsal lamellodisc 129 ± 3 (122–136, *n* = 30) in diameter; lamella 1 unpaired, forming complete ring 66 ± 2 (62–69, *n* = 30) in diameter; lamellae 2 and 3 unpaired, anteriorly closed and posteriorly with a gap (“open”), lamellae 4 to 10 paired, anteriorly and posteriorly open (Figs. 1A and 1B). Ventral lamellodisc 108 ± 5 (100–119, *n* = 30) in diameter, lamella 1 unpaired, open posteriorly 55 ± 1 (52–60, *n* = 30) in diameter with aperture 33 ± 4 (23–47, *n* = 30); lamellae 2 to 4 unpaired, open anteriorly, 5 to 10 paired, open anteriorly and posteriorly (Figs. 1A and 1C). Dorsal anchor with incipient guard (Fig. 1F), a: 32 ± 1 (30–35, *n* = 60); b: 30 ± 1 (28–33, *n* = 60); c: 17 ± 1 (16–19, *n* = 60). Two lateral dorsal bars wide lateral end; Db: 67 ± 1 (65–69, *n* = 60). Ventral anchor with developed handle (Fig. 1G), wide guard and short point; a: 40 ± 1 (38–43, *n* = 60); b: 37 ± 1 (36–38, *n* = 60); c: 33 ± 1 (31–35, *n* = 60); d: 10 ± 1 (8–12, *n* = 59); f: 19 ± 1 (18–21, *n* = 60); g: 14 ± 1 (11–15, *n* = 60). Long and thin ventral median bar (Fig. 1E) Vb: 119 ± 2 (116–123, *n* = 30), with a pointed and slightly curved end. Fourteen similar marginal hooks (seven pairs), three ventral and two dorsal on lateral haptoral lobes; two ventral pairs between anchors: H: 10 ± 1 (9–12, *n* = 60) (Fig. 1H).

Cephalic glands on each lateral side of the pharynx, both with ducts leading to three lateral head organs. Two pairs of dorsal ocelli, anterior pair in scattered pigment granules, closer together with smaller eyes than posterior pair, posterior pair with more condensed granules (Fig. 1A). Mouth sub-terminal and ventral in cephalic region. Pharynx spherical 78 ± 10 (60–93, *n* = 24) in diameter, located on midline, posterior to eyespots. Oesophagus present. Lateral oesophageal glands present. Two intestinal caeca simple, terminate blindly before haptoral peduncle. Testis subspherical, intercaecal, in anterior region of posterior half of body. Vas deferens originating on left side of testis, not encircling sinistral caecum, and enlarging to form one seminal vesicle. Prostatic reservoir pyriform, sometimes with two distinct regions, anterior to male copulatory organ.

Male copulatory organ (MCO) consists of two parts (Figs. 1I, 2), one well sclerotised and of constant shape, other slightly sclerotised and variable in appearance. Constant shape part 35 ± 1 (34–38, *n* = 30) long with a developed hook at one extremity 11 ± 1 (10–13, *n* = 30) long (Figs. 1I and 2). Variably shaped part 71 ± 5 (53–79, *n* = 30) long in total, and consists of two sections; first section large and inconsistently shaped, and attached to constant part of MCO (Fig. 2a); second section, 36 ± 1 (34–39, *n* = 30) long, generally elongate and tongue-shaped, with a more pointed appearance. An oval sclerotised rough plate, 16 ± 1 (15–18, *n* = 30) long, 10 ± 1 (9–12, *n* = 30) wide, present posterior to the MCO near genital aperture. Ovary median, subequatorial, anterior to testis, looping dorso-ventrally around right intestinal caecum. Ootype base surrounded by Mehlis glands. Vagina funnel-shaped enlarged on seminal chamber; vaginal aperture sinistral and connected with narrow duct to globular seminal receptacle, sinistrally anterior to ovary. Vitelline follicles lateral, coextensive with intestinal caeca, absent in medial region of reproductive organs, confluent anterior to MCO and posterior to testis. Eggs not observed.

**Figure 2:**
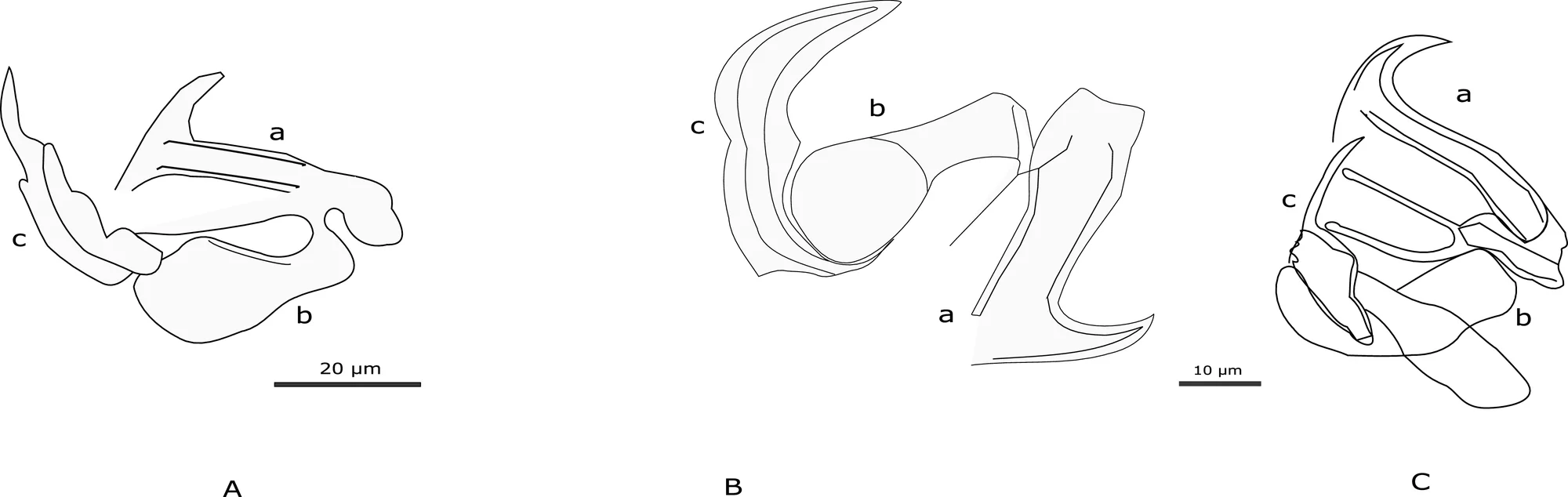
Drawings of male copulatory organs of several specimens of *L. louiseuzeti* n. sp., (A, B and C) illustrating two parts, one constant and well sclerotised (a), and the other one slightly sclerotised and variable in appearance (b, c). Scale bars: A = 20 μm; B–C = 10 μm.

#### Remarks

The morphological asymmetry of the ventral and dorsal lamellodiscs is unique among members of Lamellodiscinae. However, the morphology of the lamellodiscs varies substantially between lamellodiscine genera. Members of *Lamellodiscus*, with the type species *L. typicus*, collected from *Acanthopagrus australis* (Günther, 1859) (Sparidae) in Australia, present ventral and dorsal lamellodiscs with the intermediate lamella incomplete, partially fused, or free at the midline [31]. Members of *Telegamatrix* with the type-species *T. pellona* Ramalingam, 1955 described from *Ilisha melastoma* (Bloch & Schneider, 1801) (Pristigastridae) in India, have two identical lamellodiscs with seven to ten “B”-shaped cuticularised lamellae [61]. Members of *Calydiscoides,* with the type-species *C. australis* Young, 1969 infecting *Lethrinus miniatus* (Forster, 1801) (Lethrinidae) from off New Caledonia, South Pacific, have two identical lamellodiscs (dorsal and ventral) made up of ten unpaired lamellae forming telescopic tubes. Members of *Protolamellodiscus*, with the type-species *P. serranelli* [23, 54] infecting *Serranus cabrilla* (Linnaeus, 1758) (Serranidae) off Sète (France), have identical lamellodiscs composed of seven to ten lamellae, with lamellae 1 and 2 forming a closed circle, while the others are more or less open anteriorly [23, 54]. Finally, *Lamellodiscus echeneis*, see [15], a parasite of *Sparus aurata* Linnaeus, 1758 (Sparidae) from France, has a single ventral lamellodisc, whose first lamella is closed [22]. Therefore, this new asymmetric configuration of the lamellodiscs is not unexpected. Furthermore, while the currently known 61 species of *Lamellodiscus* resembled each other morphologically and anatomically, the sclerotised structures allow the distinction of morphological groups [19], specifically based on the morphology of squamodiscs and MCO [19]. The species described in this study is completely different from all those known to date, based on the morphology of these sclerotised structures. Similarly, the morphology of the squamodiscs does not allow the present species to be placed in any of the three described morphological groups in *Lamellodiscus* [32].

#### Phylogenetic analyses

We obtained three DNA sequences of a specimen of *L. louiseuzeti* n. sp., one sequence for each targeted DNA fragment: PZ390892 (*28S*), PZ390890 (*18S-*ITS1), and PZ387863 (*COI*). We applied ML analyses and BI to a concatenated alignment of partial *18S* rDNA, ITS1, and *COI* sequences (1,315 bp, 448 parsimony-informative sites), and a separate *28S* rDNA alignment (882 bp, 293 informative sites) of species of Lamellodiscinae, and species of Diplectaninae serving as outgroup. For the concatenated analysis, single-locus phylogenies of *18S* rDNA and ITS1 (Kendall’s W = 0.94, χ^2^ = 318, *p* < 0.05) and *18S* rDNA and *COI* mtDNA (Kendall’s W = 0.69, χ^2^ = 48, *p* < 0.05) were significantly congruent. We also found no deviations in the clades supported and overall tree topologies resulting from the ML and BI analyses. Therefore, only the ML trees are shown in Figures 3 and 4.

**Figure 3:**
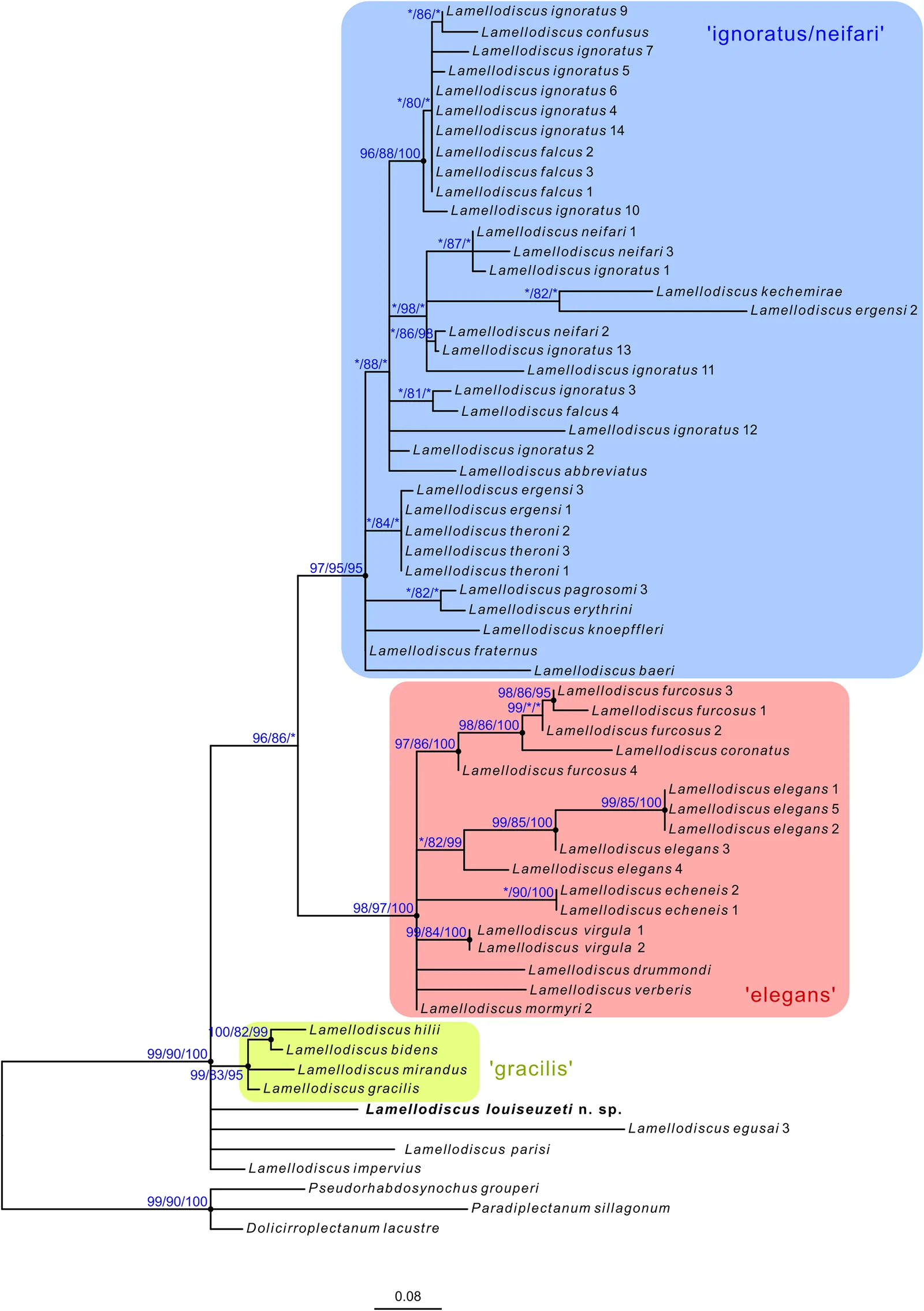
Phylogram of flatworms belonging to Lamellodiscinae (Monopisthocotyla: Diplectanidae) based on partial *18S* and ITS1 rDNA sequences as well as partial *COI* mtDNA sequences. Sequences of the species labelled in bold have been generated for this study, numbers refer to specimen IDs in Table 2. Node support: ultrafast bootstrap values (UFBoot) and Shimodaira–Hasegawa-like approximate likelihood ratios (SH-aLRT) inferred from maximum-likelihood estimation followed by Bayesian posterior probabilities (PP); asterisk (*) indicates low or moderate support below the threshold (UFBoot < 95, SH-aLRT < 80, PP < 0.95); black dots, internal nodes with strong support across all support values. Scale bar: estimated number of substitutions per site.

**Figure 4:**
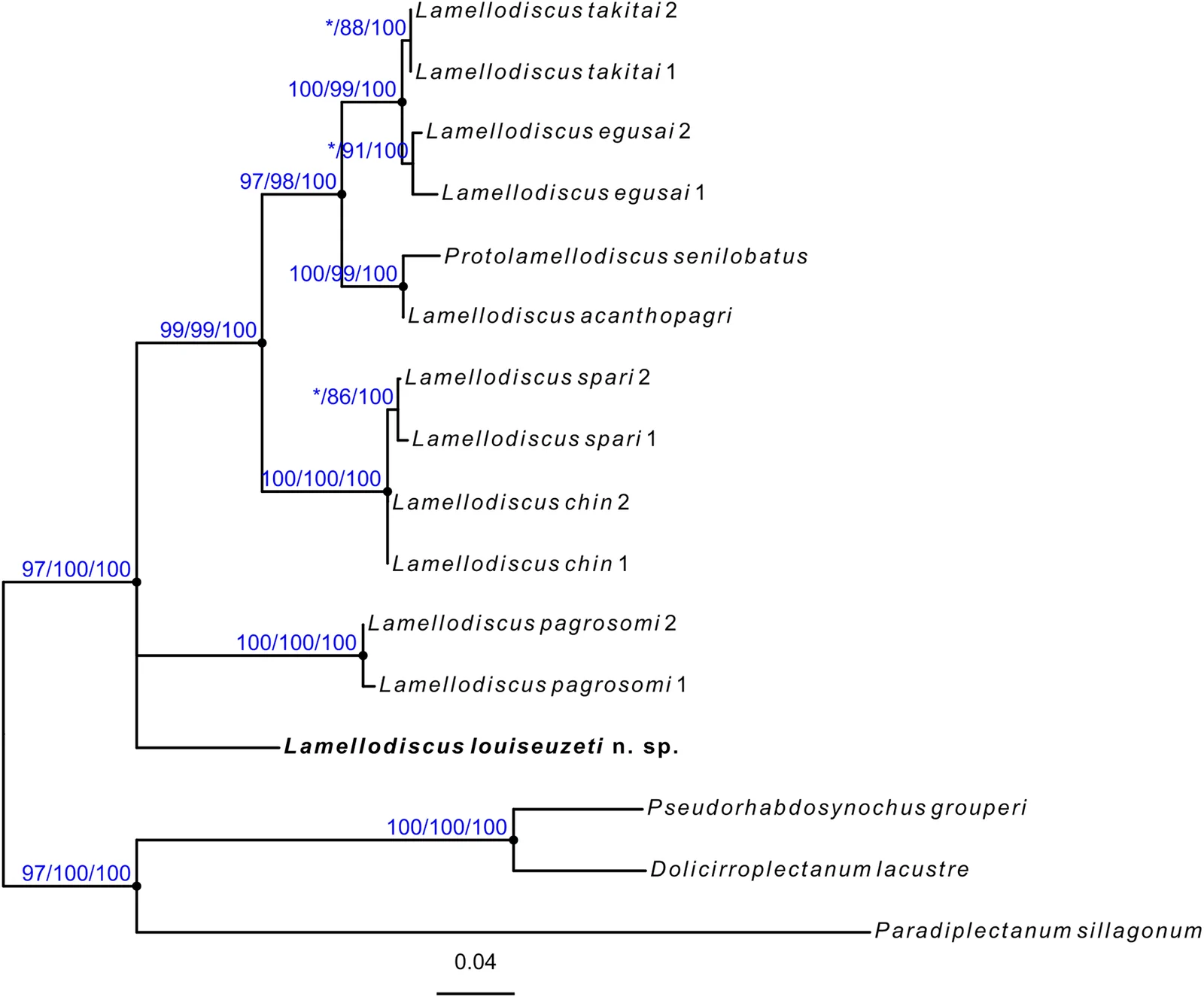
Phylogram of flatworms belonging to Lamellodiscinae (Monopisthocotyla: Diplectanidae) based on *28S* rDNA sequences. The sequence of the specimen highlighted in bold has been generated for this study. For information on node support and scale bar, see Figure 3.

The resulting concatenated tree topology shows high support for the ingroup, which includes members of *Lamellodiscus*. Three main, multi-species lineages of *Lamellodiscus* were well-supported across all support values, which we refer to here as “ignoratus/neifari” (97/95/95 for UF-Boot/SH-aLRT/PP, respectively), “elegans” (98/97/100), and “gracilis” (99/83/95) clades (Fig. 3), named after equivalent clades reported in previous studies [18, 59] (see comparison of clades in the discussion). The “ignoratus/neifari” and “elegans” clades together form a monophyletic group (96/86/*) (Fig. 3) that is well supported by the ML analysis, but less so by BI (posterior probability: 94.5). Similarly, Desdevises *et al.* [18] found support for this relationship through their maximum parsimony, but not the ML analysis of their *18S* rDNA dataset. The phylogenetic position of several species is unresolved, including that of *L. louiseuzeti* n. sp.

In two cases, sequences assigned to the same species form non-monophyletic groups. In the “ignoratus/neifari” clade, this included *L. ignoratus* Palombi, 1943, which is part of a poorly supported clade (*/80/*), which also included specimens identified as *L. confusus* Amine, Euzet & Kechemir-Issad, 2007, *L. falcus* Amine, Euzet & Kechemir-Issad, 2006*, L. neifari* Amine, Euzet & Kechemir-Issad, 2006, *L. kechemirae* Amine & Euzet, 2005, and *L. abbreviatus* Sanfilippo, 1978 that clustered with different specimens of *L. ignoratus*. Furthermore, in the “elegans” group, the only specimen of *L. coronatus* appeared to nest inside a well-supported clade (97/86/100) of specimens of *L. furcosus*.

The *28S* phylogeny included only seven species of *Lamellodiscus*, *P. senilobatus* Kritsky, Jiménez-Ruiz & Sey, 2000, and *L. louiseuzeti* n. sp., and the outgroup taxa, which limits the comparison with the analysis of the concatenated dataset. Species of *Lamellodiscus* together with *P. senilobatus* are supported as a monophyletic group (97/100/100) (Fig. 4). In the tree built from the *28S* rDNA, the phylogenetic position of the new species is unresolved, as in the tree obtained from the concatenated alignment. There are two well-supported clades including more than one species of Lamellodiscinae, with *L. acanthopagri* Roubal, 1981, *L. egusai* Nitta, 2021, *L. takitai* Ogawa & Egusa, 1978, and *P. senilobatus* in one clade (97/98/100), and *L. chin* Nitta, 2021 and *L. spari* Zhukov, 1970 in the other (100/100/100). Therefore, *Lamellodiscus* is rendered paraphyletic by the inclusion of *P. senilobatus*.

## Discussion

### Morphological evolution of members of Lamellodiscinae

Distinguishing groups among Lamellodiscinae, particularly species of *Lamellodiscus*, based on the differences observed in the morphology of the lamellodiscs is challenging, as previously reported [19]. To address this challenge and to obtain a better understanding of the evolutionary history of Lamellodiscinae, we performed a new phylogenetic analysis of this group following the description of a new species of *Lamellodiscus*, characterised by the first lamella of the ventral lamellodisc being open anteriorly, a so far unique character state. We employed a multi-marker phylogenetic reconstruction including *COI* mitochondrial DNA sequence data.

Our phylogenetic analyses showed two cases where species of *Lamellodiscus* appeared as non-monophyletic groups. In the case of *L. ignoratus*, this result may be explained by missing data, i.e. only a single specimen in our dataset had full coverage across the ribosomal DNA regions. The absence of sequence data for some DNA regions in the other specimens might explain the low support in the clade involving *L. ignoratus* and its relatives (“ignoratus” group, *sensu* [18]; part of the “ignoratus/neifari” clade in the present study). In the second case, a clade encompassing *L. furcosus* and *L. coronatus* had overall high support. Specimens of this clade also did not cluster according to data gaps in the regions included in the phylogenetic analysis. Instead, as previously noted [59], the single *L. furcosus* sequenced by Desdevises *et al.* [18] formed the sister lineage to the individuals of *L. furcosus* sequenced by Poisot *et al.* [59] and the individual of *L. coronatus*. Therefore, assuming these sequences were assigned correctly, *L. furcosus* may constitute two distinct lineages, with one of these lineages being more closely related to *L. coronatus* than to the other lineage of *L. furcosus*.

The potential presence of non-monophyletic groups of specimens carrying the same species names may indicate discrepancies between the morphological characteristics of congeneric species and their evolutionary relatedness. In this context, previous studies showed that pairwise genetic distances between sequences of specimens from the same species of *Lamellodiscus* here exceeded the levels of intraspecific differences found in other monopisthocotylan genera, such as for *L. elegans* [59]. Three factors may explain the putative high ability for diversification in monopisthocotylans, reflected in the high diversity of both DNA sequences and morphological characters observed in *Lamellodiscus*. Firstly, habitat heterogeneity is likely to be greater for small-bodied organisms (such as *Lamellodiscus*), thus favouring their diversification [24, 45, 48]. Secondly, monopisthocotylans have a direct life cycle. Life-cycle complexity has been suggested to decrease speciation rates in parasites [21]. Finally, *Lamellodiscus* appears to be composed of many species with a wide host range, which has been suggested to add molecular variability and potential for speciation [19, 20, 47, 74].

Traditionally, three morphological groups of *Lamellodiscus* are distinguished. The “ignoratus” group is characterised by identical lamellodiscs composed of ten concentric lamellae with lamella 1 circular and closed, and lamellae 2–10 opened anteriorly [54]. The “elegans” group features identical lamellodiscs composed of ten concentric lamellae with lamella 1 closed, lamellae 2–9 opened anteriorly, and lamella 10 opened anteriorly and closed posteriorly [54]. The “tubulicornis” group possesses identical lamellodiscs composed of ten concentric lamellae with lamella 1 circular and closed, lamella 2 open posteriorly and lamellae 3–9 open anteriorly and posteriorly, and lamella 10 opened anteriorly only [32].

These morphological groups partially overlap with the “ignoratus/neifari”, “elegans”, and “gracilis” clades that were inferred through the present phylogenetic analyses. The “ignoratus” group corresponds to the “ignoratus/neifari” clade reported in our Figure 3 and previous phylogenetic reconstructions [18, 59]. Furthermore, the “elegans” group corresponds to the “elegans” and “gracilis” clades suggested in our Figure 3 and previous phylogenetic reconstructions [18, 59], but these clades do not form a monophyletic group. Instead, the “elegans” clade is more closely related to the “ignoratus/neifari” clade than the “gracilis” clade. Therefore, the “elegans” (morphological) group is paraphyletic, with the “elegans”-type lamellodisc morphology potentially presenting a plesiomorphic state among species of *Lamellodiscus*. Furthermore, *L. echeneis* forms part of the “elegans” clade, while it had previously been assigned to a separate, monotypic genus (*Furnestinia* Euzet & Audouin, 1959) due to its distinct lamellodisc morphology. Only a single lamellodisc is present, but its morphology is reminiscent of the “elegans”–type, with lamellae 2–9 opened anteriorly and posteriorly [22]. No member of the “tubulicornis” group has been sequenced to date.

The three clades were reported in previous phylogenetic studies of species of *Lamellodiscus* [18, 59]. In Poisot *et al.* [59], support values of these large clades were generally low, and the authors, therefore, discussed two smaller well-supported clades that form part of the “ignoratus/neifari” clade in our analysis (named after the most represented species in the clades in the present analysis). Specifically, the “ignoratus/neifari” clade includes the “ignoratus” and “ergensi” clades reported by Poisot *et al.* [59]. Desdevises *et al.* [18] reported three well-supported clades named “ignoratus”, “elegans’, and “gracilis”. The “ignoratus” clade is the same as reported in Poisot *et al.* [59]. The “elegans” and “gracilis” clades correspond to the clades of the same names in the present study. Concerning the potential monophyly of the “elegans” and “ignoratus” clades, Desdevises *et al.* [18] found support for this relationship in the maximum parsimony, but not the ML analysis of their *18S* rDNA data. The two clades supported in the *28S* phylogeny were also supported in previous studies using only *28S* rDNA data [1, 53].

### Systematics of parasites of *Chaetodon hoefleri*: *L. louiseuzeti* n. sp. and *Pseudorhabdosynochus magnisquamodiscus*

The unique arrangement of the lamellodiscs in *L. louiseuzeti* n. sp. raises the question of whether these morphological differences justify the creation of a new genus altogether. Previously, another species, *L. echeneis*, had been assigned its own monotypic genus – *Furnestinia* – due to the presence of only a single lamellodisc [22], but the genus was later synonymised with *Lamellodiscus* based on a phylogenetic study [15]. A similar situation arose when *Protolamellodiscus senilobatus* was recently sequenced as the first member of *Protolamellodiscus* [1] as the species appeared nested inside a clade of *Lamellodiscus*, but *Protolamellodiscus* was not synonymised, unlike *Furnestinia*. Previous studies had postulated a close relationship of species of *Protolamellodiscus* with species of *Calydiscoides* due to the intermediate lamellae of the lamellodisc being fused at the midline and the presence of telescoping lamellae [20, 40]. Kritsky *et al.* [40] even proposed synonymising *Protolamellodiscu*s with *Calydiscoides*. Lastly, Kritsky *et al.* [40] previously hypothesised that *Lamellodiscus* may serve as a catch-all group for lamellodiscine species without conspicuous derived characters, rendering this genus paraphyletic.

Similar cases also exist in other monopisthocotylan groups. Within the Dactylogyridae Bychowsky, 1933, *Scutogyrus* Pariselle & Euzet, 1995 was erected for its distinct attachment organ morphology [57], but members of this group (including the type species) have consistently appeared as a group nested within *Cichlidogyrus* Paperna, 1960 in phylogenetic studies [13, 49]. Furthermore, several species of *Chauhanellus* Yamaguti, 1953 appeared nested in *Hamatopeduncularia* Bychowsky & Nagibina, 1968 in phylogenetic studies [36, 70], which coincides with an ambiguous delineation of the two taxa based on their morphological characters [36]. These examples show that, in the present case, the morphology of the lamellodiscs might not necessarily be a suitable indicator of phylogenetic relationships within Lamellodiscinae, if used to group species with and without derived character states. Given these issues concerning the creation of nested genera and the fact that our phylogenetic analyses did not resolve whether *L. louiseuzeti* n. sp. constitutes a lineage distinct from *Lamellodiscus* or is nested inside this group, we opted to consider *L. louiseuzeti* n. sp. a member of *Lamellodiscus*.

Lastly, one other monopisthocotylan species has been reported from *C. hoefleri* before: *Pseudorhabdosynochus magnisquamodiscus* (Aljoshkina, 1984), reported on the gills of a host caught off the coast of the region of “south-west Africa” (Russian: “юго-запад Африки”) [2] (no exact location was provided). We have never observed this parasite on *C. hoefleri*. Therefore, we cannot exclude that the hosts may have been misidentified, as members of *Pseudorhabdosynochus* are mainly described from fishes belonging to Serranidae Swainson, 1839. This potential misidentification has previously been suggested by Chaabane *et al.* [9].

### New morphological group: Heterolamellae

The morphology of the lamellodiscs in *L. louiseuzeti* n. sp. differs substantially from all previously described groups within Lamellodiscinae. Because the dorsal and ventral lamellodiscs exhibit distinct anatomical configurations that do not fit into the three known morphological groups of *Lamellodiscus*, we propose the creation of a new morphological group: Heterolamellae, with *L. louiseuzeti* n. sp. as its only currently known member.

- Dorsal lamellodisc: Lamella 1 forms a complete ring; lamellae 2 and 3 are closed anteriorly and open posteriorly; lamellae 4–10 are open both anteriorly and posteriorly.
- The arrangement of lamellae is asymmetrical.
- Ventral lamellodisc: Smaller than the dorsal disc. Lamella 1 is posteriorly open with an aperture; lamellae 2 and 4 are open anteriorly; lamellae 5–10 are open both anteriorly and posteriorly.

## Concluding remarks

With 61 described species and 33 sequenced so far, our knowledge of the diversity and evolution of *Lamellodiscus* remains far from complete. Despite the progress made in the previous decade to obtain molecular data from these species [18, 20, 59], these efforts have mostly focused on understanding the phylogenetic position of species or grasping the relationship between morphological and molecular evolution. In recent years, advances in genomic and transcriptomic techniques have enabled researchers to study the evolutionary history of genes that are relevant for functional adaptations in other groups of monopisthocotylans, including species of *Cichlidogyrus* [14], *Gyrodactylus* von Nordmann, 1832 [27], and *Scutogyrus* as well as *Rhabdosynochus* Mizelle & Blatz, 1941 [8], improving our understanding of the ecology and adaptive evolution of these parasites. We suggest that these techniques could be applied to species of Lamellodiscinae in future research to better understand the evolutionary history of this lineage of flatworms. For instance, the non-monophyly of *L. ignoratus* and *L. furcosus* reported here may be rooted in ecological adaptations to similar environments. Such links could be tested with the mentioned molecular tools.
